# Approaches to the Evaluation of Competency-Based Health Professions Education Implementation from Around the Globe

**DOI:** 10.5334/pme.2628

**Published:** 2026-09-29

**Authors:** Andrew K. Hall, Dorothy Andriole, Aleda M. H. Chen, Robert Englander, David Rojas, Michael S. Ryan, Christy K. Boscardin, Holly Caretta-Weyer, Timothy R. Dalseg, Ian W. Incoll, Roberta I. Ladenheim, Leila Niemi-Murola, Anna Oswald, Severin Pinilla, David A. Turner, Mary Ellen J. Goldhamer, As members of the International Competency-Based Health Professions Educators Collaborative

**Affiliations:** 1 Department of Emergency Medicine at the University of Ottawa, Canada; 2 Royal College of Physicians and Surgeons of Canada, Canada; 3 Medical Education Research, Association of American Medical Colleges, Washington, DC, USA; 4 University of Arkansas for Medical Sciences in Little Rock, Arkansas, USA; 5 Department of Medical Education at the University of Illinois College of Medicine, Chicago, Illinois, USA; 6 Temerty Faculty of Medicine, University of Toronto, Toronto, Canada; 7 University of Virginia, Charlottesville, VA, USA; 8 Maastricht University, Maastricht, The Netherlands; 9 Department of Medicine, University of California San Francisco, San Francisco, USA; 10 Stanford University School of Medicine, Palo Alto, California, USA; 11 University of Toronto, Toronto, Canada; 12 Royal College of Physicians and Surgeons of Canada, Ottawa, Canada; 13 University of Newcastle, Australia; 14 University of Melbourne, Australia; 15 Universidad Hospital Italiano de Buenos Aires, Buenos Aires, Argentina; 16 University of Helsinki, Finland; 17 Helsinki University Hospital, Finland; 18 University of Alberta, Edmonton, Canada; 19 University Hospital of Old Age Psychiatry and Psychotherapy, University of Bern, Bern, Switzerland; 20 Innovation and Competency-Based Education at the American Board of Pediatrics, Chapel Hill, NC, USA; 21 Division of General Internal Medicine, Massachusetts General Hospital, USA; 22 Harvard Kidney Urology Hematology Training Institute, Boston, Massachusetts, USA

## Abstract

Competency-based health professions education (CBHPE) is in various stages of implementation around the globe. While program evaluation is essential to drive improvement, adaptation, and the evolution of CBHPE, it may be intimidating for educators given the breadth of program evaluation methods and wide variation globally in local resources and expertise. In this manuscript, the authors summarize key examples of program evaluations highlighting an array of CBHPE implementations, evaluation methods, and settings. The examples are organized by five evaluation focus areas: readiness to implement, fidelity of implementation, assessment program validity, educational outcomes, and clinical outcomes. Illustrative examples from around the globe provide a roadmap featuring program evaluation methods that may be adapted to the setting, goals, size, and resources of individual programs. Capturing the evaluation of CBHPE implementation is essential for informing ongoing implementation efforts and iterative improvements to CBHPE.

## Introduction

Competency-based medical education (CBME) and competency-based health professions education (CBHPE), herein referred to collectively as CBHPE, are in varying stages of implementation across professions around the globe. Program evaluation is a critical component of CBHPE implementation, with evaluation findings and subsequent lessons learned driving program adaptations and the evolution of CBHPE [1,2]. While there are multiple described CBHPE program evaluation methods, the challenge of engaging in evaluation may be intimidating for educators, given the breadth of methods and wide variation globally in local resources and expertise. Further, there are limited existing syntheses of CBHPE program evaluation approaches across health professions education journals. Tracking evaluation of CBHPE implementation is further complicated by a lack of standardized search terms and extensive practical site- or setting-specific works, which may not be published in the peer-reviewed literature. Yet, ongoing evaluation of CBHPE program implementation is essential to drive evolution and adaptations in this emerging field [2,3].

The goal of this manuscript is to summarize a series of approaches to CBHPE program evaluation from around the globe organized by evaluation focus and methods chosen. We provide a narrative synthesis of evaluation efforts from a broad array of contexts identified by study authors and the International Competency-Based Health Professions Educators (ICBHPE) Collaborative. We grounded the work with Patton’s definition of evaluation science: the “systematic inquiry into the merit, worth, utility, and significance of whatever is being evaluated” [4]. The works chosen span an array of approaches, including traditional evaluation work, but also reflective program descriptions and methodologies which take a more traditional research approach. Recognizing the distinction between research and program evaluation, we ensured that all studies met the Patton definition. The evaluation projects, identified by study authors and selected via consensus, are intentionally organized into five areas of focus: readiness to implement, fidelity of implementation, assessment program validity, educational outcomes, and clinical outcomes. These five categories have been derived iteratively by the study authors through our synthesis process in reflecting on both the included studies and other CBHPE evaluation literature. These areas of focus can be conceptualized as an evaluation arc, with readiness to implement CBHPE as a potential first step, followed by fidelity of implementation studies, which may be coupled with evaluations of assessment program validity. Educational and subsequent distal clinical outcomes are typically studied later in the CBHPE implementation process, recognizing that this ideal arc and timeline are not so simply enacted. Further, we acknowledge that this arc is not necessarily linear in nature, but rather a process of co-occurrence with periods of iterative revision and adaptation in focus when evaluating a complex and transformative system change. We do not intend to describe methodological procedures for each example in depth but rather aim to illustrate how each example helps us understand the value of that evaluation approach. Approaches highlighted may include specific evaluation methodologies or frameworks which either guide or organize the evaluation. While these evaluation approaches have been provided as illustrative examples in each focus area, the approaches and methods are by no means specific to that focus; rather the methods and approaches are often transferrable across focus areas. We then summarize across the experiences of CBHPE evaluation to better define what has been done so far and identify existing gaps. Studies selected are representative of both small-scale and large-scale evaluations of CBHPE implementation from across the globe. Table 1 provides a summary of illustrative examples of evaluation studies from around the globe, organized by focus, that are described in detail in the text.

**Table 1:** Five evaluation focus areas mapped to representative examples of methods, approaches, or frameworks used to evaluate the implementation of undergraduate medical education (UME) or graduate medical education (GME) competency-based health professions education (CBHPE) programs around the globe.


EVALUATION METHOD, APPROACH, OR FRAMEWORK	SETTING FOR EXAMPLE(S) DESCRIBED	*LEVEL OF TRAINING UME, GME

**1. Readiness to Implement**		

R = MC^2^ framework	Royal College of Physicians and Surgeons of Canada, Competence by Design [9]	GME

**2. Fidelity of Implementation**		

Innovation Configuration Mapping	Royal College of Physicians and Surgeons of Canada, Competence By Design [17]	GME

Rapid Evaluation	Queen’s University, Canada [12]; Royal College of Physicians and Surgeons of Canada; [17]	GME

Realist Evaluation	University of Manitoba, Canada [24]	GME

Logic model	Association of American Medical Colleges (AAMC) Core Entrustable Professional Activities for Entering Residency (Core EPAs), 10 school pilot, U.S. [25]	UME

	College of Family Physicians of Canada – Triple C Curriculum [30]	GME

Other Fidelity-Focused Evaluation	Aga Khan University, Pakistan [31]	GME

	National program director survey, Finland [32]	GME

	Emergency Medicine, Canada CanDREAM Study [33]	GME

**3. Assessment Program Validity**		

Extrapolation Inference	Family Medicine residency program, Ambulatory Healthcare Services, Abu Dabi [39]	GME

Implications Inference	Association of American Medical Colleges (AAMC) Core Entrustable Professional Activities for Entering Residency (Core EPAs), 10 school pilot, U.S. [25,43]	UME

Comprehensive Validity Argument for Program of Assessment	Accreditation Council for Graduate Medical Education (ACGME) Milestones, U.S. [47,48]	GME

	Education in Pediatrics Across the Continuum (EPAC), U.S. [52,53]	UME, GME

**4. Educational Outcomes**		

Descriptive Evaluation	University of Toronto Orthopedic residency program [58]	GME

	Entrustable Professional Activities (EPAs) in The Netherlands [59]	GME

Stakeholder experience: Resident experience (surveys)	Resident Doctors of Canada (RDOC), Royal College of Physicians and Surgeons of Canada [60]	GME

	Resident doctors of Fédération des Médecins Résidents du Québec (FMRQ) [61]	GME

Stakeholder experience: Resident experience (interviews and focus groups)	Competence by Design: Surgical and procedure-based Canadian training programs, Canada [62]	GME

	Competence by Design: Internal Medicine residents, Canada [63]	GME

Large-scale stakeholder analyses	Promotion in Place competency-based time-variable training, U.S. [67]	GME

	Faculty of Veterinary Medicine, Utrecht University, The Netherlands [68]	UME

Qualitative educational case study	Transitioning in Internal Medicine Education Leveraging Entrustment Scores Synthesis *(*TIMELESS) Internal Medicine at the University of Cincinnati, U.S. [69]	GME

	National Core Curriculum implemented in one medical school, Turkey [71]	UME

Mixed Methods Evaluation	Australian Orthopedic Association Training Program, Australia [79]	GME

Quantitative Outcome Evaluation	Education in Pediatrics Across the Continuum (EPAC), U.S. [51,73,94]	UME to GME

	Canadian Family Medicine Residency [76]	GME

	Pathology residency program at Massachusetts General Hospital, U.S. [77]	GME

**5. Clinical Outcomes**		

Evaluation using Objective Structured Clinical Examinations (OSCE)	The COBALIDATION Trial, Spain [83]	GME


*Level of training: Undergraduate Medical Education (UME) e.g. medical or veterinary school; Graduate Medical Education (GME) e.g. residency and/or fellowship training; Competency-based Health Professions Education (CBHPE).

### Evaluation Focus Area 1: Readiness to Implement

The adoption of CBHPE has historically been characterized as a transformative change necessitating a significant shift in behavior for all involved [5]. Understanding the readiness for a program or system to change is an important first step to implementing CBHPE [6]. Through this understanding, programs can better prepare for successful CBHPE implementation with targeted intervention prior to actual implementation. While there are terms and tools to measure organizational readiness described in the business and health services literature [7], there have been questions raised regarding the validity and reliability of these measurement tools in an educational paradigm [6]. Subsequently, there is a paucity of literature describing the measurement of readiness to implement CBHPE, with only one key illustrative example included in this manuscript.

**R = MC2 framework:** The R = MC^2^ framework [8] which describes three distinct components: organizational *motivation* to implement an innovation (beliefs about and support for an innovation), the *general capacity* of an organization (context, culture, current infrastructure, and processes in the organization), and the *innovation-specific capacities* required for an innovation *(resources, technical expertise, infrastructure)*. Cheung et al employed a survey informed by the R = MC^2^ framework, which was disseminated to all program directors in disciplines representative of the specialist medical and surgical residency training programs directed by the Royal College of Physicians and Surgeons of Canada during the implementation of Competence-By-Design (CBD) [9]. For this multi-specialty evaluation, little difference was found in overall readiness between programs; there was a positive correlation between all three readiness components, suggesting that interactive and reciprocal processes may exist. The authors ultimately highlight the importance of ensuring that stakeholders understand the rationale for implementing CBHPE as well as its defining concepts and processes.

### Evaluation Focus 2: Fidelity of Implementation

The degree to which CBHPE is implemented as intended, known as fidelity of implementation, is a critical early evaluation focus [10,11]. Understanding the fidelity of implementation can guide designers in modifications to both implementation strategies and elements of the program, and provide timely feedback regarding which elements of CBHPE innovations are working or not [12]. Further, measuring the fidelity of implementation is needed before outcomes are measured and conclusions are drawn regarding the impacts of a program [10]. These types of utilization-focused evaluations [13] are common across medical education innovation evaluation and often result in important early program adaptations. The evaluation frameworks and methodology examples described in this section are not necessarily specific to fidelity of implementation and may be utilized to represent additional areas of focus. Some do, however, have a particular alignment with identifying successes and challenges with fidelity of implementation, such as Innovation Configuration Mapping [14] and Rapid Evaluation [12]. While others, such as the Logic Model [15] and Realist Evaluation [16], are employed to evaluate fidelity in this example, sometimes within a broader or more holistic evaluation approach.

**Innovation Configuration Mapping:** Innovation configuration mapping facilitates delineation of each implementation component, including a description of the spectrum of observable variations expected for that component, ranging from idealized implementation to non-implementation [14]. The Pulse Check study by the Royal College of Physicians and Surgeons of Canada [17] used innovation configuration mapping to first identify the key components of their CBHPE implementation and then conduct a national annual survey of program directors to measure the degree of implementation of each component. This study identified that while some elements of CBD were implemented as intended, such as competence committees and Entrustable Professional Activities (EPA) assessments, other key components, such as effective coaching in the moment, coaching over time, and individualized learning plans, were not [17].

**Rapid Evaluation:** Rapid Evaluation is an approach in which intended innovation is defined first, then actual implementation is compared to intended implementation, and data-informed program adaptations are made [12,18]. Hall and colleagues took this approach, employing focus groups and interviews across multiple disciplines and programs, to better understand the stakeholder experience early in CBD implementation [12,17]. Their findings helped identify significant frustrations related to assessment of both front-line faculty and trainees and the need for both longitudinal and workplace-based assessment processes. These results informed the revised CBD 2.0 [19], which provided more flexibility to program-level assessment processes and a renewed focus on coaching.

In addition to the evaluation work from the Royal College of Physicians and Surgeons of Canada, several other institution-level fidelity-focused evaluation strategies have featured prominently. At Queen’s University, an institution-wide approach identified challenges across programs associated with assessment burden and variable experiences with EPA assessments and feedback [20]. These evaluation studies drove program-specific revisions [21,22] and institution-level adaptations, particularly for assessment processes [23].

**Realist Evaluation:** Realist Evaluation starts with an initial program theory and then asks the question, what works for whom, in which context and circumstances, and why, to understand the Context + Mechanism = Outcome (CMO) configurations of the innovation [16]. Realist evaluation provides the unique ability to contribute to an understanding of why evaluations of the same CBHPE model or design implemented in different contexts may yield different findings. At the University of Manitoba, a realist evaluation strategy identified multiple factors that contributed to or detracted from effective CBHPE implementation, noting the negative impact of the simultaneous implementation of an electronic curricular management system and CBHPE systems on resultant fidelity of implementation [24].

**Logic Model:** A logic model provides a coherent framework for aligning implementation activities with evaluation measures, supporting iterative refinement throughout the pilot and enabling a structured assessment of feasibility, fidelity, and outcomes over time [25,26,27]. In the United States (U.S.), the Association of American Medical Colleges (AAMC) employed a detailed logic model to outline the inputs, activities, expected outputs and outcomes anticipated in their implementation of the Core Entrustable Professional Activities for Entering Residency (Core EPAs) [15,25,28]. They engaged in a multi-institutional evaluation with a primary goal of examining the feasibility and fidelity of implementation of this framework across 10 diverse medical schools [29]. Five targets were prioritized for measurement, specifically two measures of fidelity (student awareness of the Core EPAs and the validity and reliability of supervisory/co-activity scales), and three outcomes (summative entrustment decisions for each Core EPA, confidence in these entrustment decisions, and preparedness of graduates for residency). Multiple data sources were mapped to these measures, including national survey data, pilot-specific surveys, entrustment decision data, and single- and multi-institutional studies.

An additional prominent use of the Logic Model in a theory-based evaluation is that performed longitudinally by the College of Family Physicians of Canada in the evaluation of the Triple-C Competency-based Curriculum [30]. Here, program theory was developed early as part of implementation in the form of a detailed logic model, which then formed the foundation of, and key focus areas for, a process and outcome evaluation. This approach identified areas supporting implementation of CBHPE with fidelity, and areas in need of improvement, including enhanced support structures and planning.

**Other Fidelity-Focused Evaluations:** In another important example of fidelity-focused evaluation, Riaz et al. [31] examined the implementation of CBHPE at Aga Khan University in Pakistan across five residency programs. Evaluation was conducted 18 months post-implementation using qualitative focus group discussions with program directors to explore perceptions of fidelity, barriers, facilitators, and early impacts on assessment, learning, and governance. Successes and challenges were identified in the areas of assessment and feedback and informed ongoing implementation efforts.

In Finland, a national competency-based residency training reform was launched in the year 2020 to replace the traditional time-based model, which had little tradition of formative assessment. After five years, a national survey of program directors showed major variation across universities and specialties in assessment, supervision quality, and mentoring resources. Although all specialties had constructed and implemented EPAs, this study identified the need for more consistent national structures and harmonized educational practices [32].

Seeking to understand the early features of an EPA assessment program implemented in Emergency Medicine in Canada, the CanDREAM study [33] created a specialty-specific database of program-level EPA assessments across training programs. Significant variation in EPA-based assessment numbers and promotion timelines across programs was found, as well as significant differences when compared to national guidelines, informing both local and national-level revisions.

### Evaluation Focus 3: Assessment Program Validity

Competency-based advancement and graduation decisions in CBHPE programs are informed by programs of assessment that include multiple methods and assessors within educational systems [34]. A program of assessment is one of the core components of CBHPE [5] and studies have sought to gather validity evidence supporting their use in higher-stakes progression decisions [34,35]. These evaluations, while often part of the overall evaluation of fidelity of implementation, can be viewed as a distinct focus of evaluation given the unique theories, frameworks, and processes involved. Messick has defined validity as “an evaluative summary of both the evidence for and the actual—as well as potential—consequences of score interpretation and use” [36]. Others have described approaches to validity arguments [37] and discussions of what validity means in a CBHPE program of assessment utilizing entrustment [38]. In this section, we limit our description to a few examples of evaluation studies whose main focus was the validity of assessment tools or systems organized by two main inferences (extrapolation and implications) using Kane’s Validity Framework [37] as well as examples of a comprehensive validity argument. Kane’s scoring and generalization inferences are under-represented in the CBHPE evaluation literature.

**Extrapolation Inference:** In the Family Medicine residency program of the Ambulatory Healthcare Services in Abu Dhabi, a comparison of trainee early assessment data (pre-CBHPE) and Milestones data post CBHPE implementation with subsequent graduating in-training exam performance found a much stronger correlation for the Milestone data than pre-CBHPE assessment data, supporting the extrapolation inference for validity [39]. Studies focusing on psychometric properties are beneficial to establish validity evidence for competency-based programs of assessment.

**Implications Inference:** Multiple studies have examined the validity and reliability of assessment scales and processes in the AAMC Core EPAs Pilot [40,41,42]. Specifically, analyzing available EPA assessment data and entrustment decision-making outcomes by looking at the percentage of students for whom an entrustment decision could be made in a large pooled sample, Brown et al. [43], demonstrated that medical school-level interventions in the Core EPA Pilot could improve the quality and extent of data available to competency committees to inform entrustment decision-making. As another example, Amiel et al. [25] described the collection of the multiple sources of assessment and decision data, including trainee self-reported frequency of observation/feedback in Core EPAs and readiness to perform Core EPAs under indirect supervision, numbers of WBAs completed, trained faculty groups’ entrustment decisions for graduating students, and program directors’ assessments of graduates’ preparedness for residency. Medical schools participating in the Core EPAs pilot made progress in building robust programs of assessment while identifying important gaps in evidence for the validity of graduation decisions.

**Comprehensive Validity Argument for Program of Assessment:** In the U.S., Accreditation Council for Graduate Medical Education (ACGME) Milestones function as narrative descriptors of competencies within six core competency domains [44] providing a developmental roadmap of the specialty-specific knowledge, skills, and behaviors that residents must demonstrate to achieve competence and readiness for independent practice [45,46]. Focused on scoring, generalization, and extrapolation inferences, the ACGME has evaluated the validity of the bi-annual Milestone ratings submitted by all accredited residency and fellowship programs and based on national-level aggregation, concluded that Milestone evaluations reliably assess outcome competencies [47,48,49,50].

The Education in Pediatrics Across the Continuum (EPAC) U.S. pilot was designed to assess the ability to advance learners from Undergraduate Medical Education through Graduate Medical Education in a time-variable manner [51]. This project involved longitudinal assessment of learners across the continuum with a program of assessment comprised of workplace-based assessments, summative assessments, learner-driven individualized learning plans and quarterly competency committee reviews [52,53]. The authors found that learning curves followed Thurstone’s theory of negative exponential growth, providing evidence of construct validity [52]. This program of assessment was grounded in the EPA framework and demonstrated strong validity evidence for time-variable promotion within and across medical education contexts, particularly relating to the extrapolation and implications inferences. In addition to the EPAC pilot, multiple other large investigations in pediatric GME in the U.S. have demonstrated validity evidence to support integrating EPA-based assessment into competency committee processes [54,55,56].

### Evaluation Focus 4: Educational Outcomes

There have been broad calls for impact and outcome evaluation relating to CBHPE from the medical education community [3,10], but significant challenges in their meaningful measurement remain. A proposed logic model for CBHPE [10] identifies many potentially measurable downstream impacts on educational outcomes such as enhanced quality of feedback, earlier identification of trainees requiring remediation, enhanced readiness for practice, alignment of medical education with community health needs, and enhanced patient care outcomes. However, implementing CBHPE with fidelity has been challenging, and thus most implementers have yet to consider the evaluation of more distal outcomes [3]. Because distal clinical outcomes have posed significant barriers to measurement and become entangled with multiple care team members, outcomes evaluation has often focused on more proximal educational outcomes, including individual student or trainee outcomes (micro), the program (meso), or the system (macro) [57]. In this section, we highlight approaches and evaluation methods that focus on evaluating educational outcomes over the course of CBHPE program implementation, providing key examples to inform the implementation and evaluation plans for both new and ongoing programs. Of note, several examples also included elements of focus related to fidelity of implementation. This highlights that there are often multiple goals of evaluation efforts, and overlap may exist between fidelity of implementation and early educational outcomes. Studies were included here if their focus was primarily on educational outcomes, understanding that some evaluation approaches overlapped and included aspects of fidelity of implementation as well.

**Descriptive evaluation:** The University of Toronto Orthopedic residency program was the first time-variable surgical training program in North America [58]. While a specific evaluation framework was not used, program leadership described a critical and reflective evaluation of the program 8 years post-implementation. They report on successes, challenges, and processes that resulted in an increase in resident assessments by 3–5-fold. Further, all residents passed their board examinations on first attempt. Many participating residents were deemed competent for early graduation up to one year prior to the traditional standard graduation date.

Another example of a descriptive reflective evaluation in residency training focused on the implementation of EPAs in the Netherlands [59]. Authors describe the rationale and process of implementing CBHPE across 30 training programs in the Netherlands, and describe educational outcomes relating to time-variability, shortening the average length of training by 3 months.

**Stakeholder Experience:** The resident CBHPE experience has been closely monitored during the roll-out of CBD in Canada. In a prominent collaboration between the Resident Doctors of Canada and the Royal College of Physicians and Surgeons of Canada, residents were surveyed every other year (2021, 2023, 2025) using national jointly developed questionnaires. These surveys identified a strong signal of negative impact of CBD on trainee wellness, due to perceived assessment burden, difficulty getting faculty to complete assessments, and threats of non-progression in training due to incomplete assessment data [17,60]. In addition, highly influential stakeholder experience surveys have been conducted by the Fédération des Médecins Résidents du Québec (FMRQ) regarding resident doctors identifying threats to effective feedback due to overly performance-focused assessment processes and progression decision-making processes without perceived educational benefits [61]. Further, early in Canada’s CBD implementation, semi-structured interviews and focus groups evaluated the resident experience with EPA assessment in surgical and procedure-based training programs [62], identifying trainees’ perception of assessment burden in these specific disciplines.

A focus group study of internal medicine residents provided feedback on the barriers and facilitators to using EPA assessments during CBD implementation [63].

**Large Scale Stakeholder Analysis:** As CBHPE programs are implemented, stakeholder-focused program evaluation, known as stakeholder analysis, can provide critical insights and perspectives of the many invested partners and collaborators within the education system to inform continuous program improvement [64,65]. It is important to note that in the CBHPE literature, resident and program director perspectives dominate, while patient and community perspectives are less common, highlighting both a gap and an opportunity in current evaluation efforts.

Promotion in Place, a competency-based time-variable residency-training pilot in the U.S., sought stakeholder input regarding the value of a specific model that included a period of “sheltered independence” in their department as attendings after qualifying residents graduated early [66]. Semi-structured interviews with both participating and non-participating residency programs included residents, program directors, clinical leaders, and members of national medical education organizations [67]. Participants noted the potential for enhancing trainee satisfaction and wellbeing, and the appeal of a period of “sheltered independence” as a transition to practice, by promoting independent decision-making and enhancing confidence.

Stakeholder analysis in CBHPE has also been explored in the veterinary medicine context. In a programmatic assessment initiative in the Faculty of Veterinary Medicine, Utrecht University, Bok and colleagues [68] examined perceptions of students, clinical supervisors, and assessment committee members during the rollout of workplace-based assessments. Focus groups highlighted students’ perceptions that formative assessments felt summative, clinical supervisor uncertainty about the use of their ratings and feedback, and challenges with the quality and feasibility of feedback within busy clinical environments. Collectively, these stakeholder analysis program evaluations emphasize the value of capturing broad stakeholder experience of CBHPE implementation across programs and specialties.

**Qualitative educational case study:** Qualitative lines of inquiry can provide robust richness for educational outcomes of CBHPE, highlighting findings that offer transferability to other settings. We provide two examples of qualitative educational case studies.

Transitioning in Internal Medicine Education Leveraging Entrustment Scores Synthesis *(*TIMELESS) was implemented as a time variable residency training model in Internal Medicine at the University of Cincinnati in the US. Program leaders used qualitative educational case study methodology and conducted a series of longitudinal resident interviews over 3 years to understand the program impact on resident motivation for learning, assessment, and feedback [69]. They found TIMELESS residents were more motivated to learn and seek feedback compared to residents in the standard program, though some TIMELESS residents reported a tension between a performance and growth mindset.

Turkey’s National Core Curriculum and the implementation of CBHPE in one medical school provides another example of qualitative educational case study, using the Context, Input, Process, and Product evaluation (CIPP) model [70,71]. Semi-structured interviews with faculty and students revealed inconsistencies between CBHPE theory and practical implementation of curricular frameworks. Stakeholders agreed that iterative cycles of improvement and curriculum evaluation were needed to refine and fully implement competency-based training.

**Quantitative Outcome Evaluation:** Quantitative lines of inquiry can also provide meaningful insights for CBHPE educational outcomes, highlighting findings that offer generalizability insights.

EPAC is a competency-based time-variable model of training implemented at four U.S. sites where advancement through medical school and pediatric residency followed by fellowship or practice is based on demonstrated competency [72]. In this model, progression from medical school to residency was based on the Association of American Medical College’s (AAMC) Core EPAs for Entering Residency [29], and progression into fellowship or independent practice was based on competency in the General Pediatrics EPAs. The primary outcome of feasibility of time-variable competency-based advancement was measured by transition time from medical school to residency, and then residency to independent practice transition points [73]. At both transition points, participants showed a range of time for readiness, but almost all participants were ready for the respective transitions ahead of their fixed-time counterparts in the same programs. Student performance in the Core EPAs was plotted on growth curves and demonstrated progressive learner competency acquisition and EPA supervision level ratings from program entry through medical school graduation [51]. Additional quantitative outcomes for pediatric residents included Milestones ratings, first-time pass rates for the American Board of Pediatrics certifying examination, and attainment of fellowship or job placements after graduation; all of which demonstrated that EPAC participants were at or above the level of students in the traditional time-fixed program from the same residency [73]. EPAC has, in addition, been evaluated using qualitative methods, to better understand medical student perspectives [74] and subsequent choice of pediatrics as a career [75].

Another quantitative evaluation is from Canadian Family Medicine residency training, which aimed to measure the impact of CBHPE implementation on remediation patterns. In this large-scale retrospective cohort study including more than 400 family medicine residents, evaluators found that after implementation of the Triple-C Competency-Based Curriculum there was improved identification of struggling residents and enhanced program abilities to remediate individual trainee competency deficiencies [76].

Lastly, the Promotion in Place program measured acceptability, feasibility, and educational outcomes such as time to competency achievement, board pass rates, Milestones, and safety reports [77]. This simple evaluation approach demonstrated non-inferiority between residents who qualified for early residency graduation and a period of “sheltered independence” as attendings, as compared to residents who remained in the standard training program.

These quantitative studies underscore the importance of evaluating educational outcomes in both pilots and established programs [64]. Studying educational outcomes has been prioritized in several settings [57,78], as these outcomes are often a more feasible target for evaluating the impact of educational innovations. Further, evaluating educational outcomes while implementing CBHPE offers the opportunity for real-time continuous program improvement.

**Mixed Methods Evaluation:** If feasible, mixed methods approaches that employ qualitative and quantitative approaches can provide the deepest insights into CBHPE educational outcomes.

In 2017, the Australian Orthopaedic Association introduced a competency-based orthopaedic surgical training program, replacing the traditional time-based model [79]. Its evaluation, guided by the Core Components Framework [5], aimed to assess goal attainment and identify gaps in curriculum, assessment, and supervision. Using a mixed-methods approach, the evaluation analyzed trainee portfolio data, surveys, and focus groups. Findings indicated improved competence progression and examination outcomes but highlighted challenges such as inconsistent delivery of professional competencies, assessment burden, and limited stakeholder engagement. This evaluation emphasized the need for streamlined assessment processes and enhanced faculty development to sustain implementation.

### Evaluation Focus 5: Clinical Outcomes

The imperative to measure the impact of CBHPE implementations on clinical outcomes has been called for across settings and groups [10,57]. In fact, enhanced healthcare provider performance and resultant improvement in patient care are the ultimate goals of CBHPE implementation [80]. Previous studies have demonstrated that the nature of medical training has a significant impact on downstream clinical outcomes [81], but these studies are few and face significant challenges [82]. Nonetheless, evaluating clinical outcomes remains central to understanding the return on investment for the time and resources demanded for the implementation of CBHPE. Below we highlight one of the few program evaluation studies that has yielded a link to clinically relevant outcomes, despite being measured in the simulation environment. Further, we identify other work that correlates in-training assessments to downstream clinical outcomes. These studies, while not measuring the impact of a CBHPE program on direct clinical outcomes, highlight a first step in providing evidence that in-training intervention related to these measurements could yield significant downstream clinical outcomes.

The COBALIDATION Trial was a multicenter cluster randomized trial conducted across 13 Spanish Intensive Care Units (ICUs) that compared the performance of trainees from a newly implemented competency-based model of Intensive Care Medicine residency training known as CoBaTrICE (Competency-Based Training in Intensive Care Medicine in Europe) to Spain’s traditional time-based model for training in Intensive Care Medicine [83]. In this study, the clinical outcome measured was performance on a simulation-based Objective Structured Clinical Examination (OSCE) that assessed performance in five simulated clinical crisis scenarios; results are notable in that competency assessments between the two groups were not statistically significant. Despite the simulation environment being a surrogate for actual clinical performance, this study highlights an approach to measuring short-term (in-training) meso (program) level clinical outcome impacts [57].

In two studies from the United States, trainee physician Milestones ratings have demonstrated a correlation with subsequent clinical performance. Han et al. [84] identified that trainees with low Milestones ratings in professionalism and communication skills were at higher risk of patient complaints in their early independent practice. Further, ACGME Milestone assessment of surgical trainees has been associated with early career clinical outcomes in the discipline of Vascular Surgery [85]. These two examples highlight the potential for in-training intervention via CBHPE training processes to impact downstream clinical outcomes.

## Discussion and Future Directions

There are many approaches to evaluating the implementation and impact of CBHPE programs that align with a variety of evaluation goals and methodologies. In this article, we have presented five areas of focus for evaluation in CBHPE and selected key examples from a variety of contexts to highlight available evaluation approaches, methodologies, and findings. Importantly, we propose that the progression of focus can be conceptualized as an evaluation arc, with a progression of evidence providing grounds for iterative program revision and/or evidence of program impact. A focus on readiness to implement CBHPE aligns with the broader change management literature [86], emphasizing the importance of both organizational supports and individual motivation, capability, and opportunities [9]. Measuring fidelity of implementation is the next critical step prior to considering evaluation of outcomes. The literature here highlights that while some core components of CBHPE, such as a set of outcomes competencies and a program of assessment, are often implemented as intended, other critical features like longitudinal coaching and individualized learning plans are less consistently implemented [17]. Further, achieving a sufficient volume of assessment to inform progression and certification decisions remains a common challenge. Assessment validity must be considered at the beginning of CBHPE implementation as well: defining purpose, decision-making, and evidence to support decisions [35]. Programs should use multiple sources of evidence, while remembering that validity is not static, evolving with people, places, and time [87]. A focus on educational outcomes can offer important evidence of more proximal program impact on learners, the learning environment, and the processes of training within a program [10]. Looking directly at key stakeholder experiences provides valuable insights into CBHPE perceptions and implementation, highlighting issues such as assessment burden and learner wellness that can inform ongoing adaptation [17,23,67]. Finally, a focus on the measurement of clinical outcomes remains a challenging ‘holy grail’, with limited published efforts thus far [88,89]. Only time and sustained evaluation efforts will aid in this evaluation challenge.

Within each focus, we have highlighted examples of many evaluation methodologies and frameworks. There is no one specific method that is perfect for a given evaluation, but rather a list of options. Readers are encouraged to consider the purpose of their evaluation and choose an appropriate framework and approach. Several program evaluation guides exist which can guide readers in their choice of methodologies [1,90].

Across the highlighted evaluation studies, several key lessons emerge. Program evaluation clearly serves as a foundation for understanding what is working, what requires adjustment, and how CBHPE can continue to evolve to meet the needs of learners, educators, and the patients they serve [64]. The diversity of evaluation approaches from smaller and simpler quality improvement initiatives to complex, mixed-methods program evaluation is both expected and valuable, reflecting contextual adaptation, global resource realities, and differing stages of program maturity. Collectively, these studies contribute to a holistic and nuanced understanding of CBHPE implementation across global settings. While less evidence is available for some of the evaluation goals, particularly with respect to clinical outcomes, this would be expected at this early phase of implementation and can act as a reminder or impetus for targeted areas of future work.

One of the great challenges in CBHPE program evaluation is that a significant amount of the meaningful work exists outside of the peer-reviewed literature, including institutional and/or specialty technical reports, local quality improvement (QI) projects, presentations, and other forms of grey literature. Some prominent examples of these include ACGME Milestone national reports [48] and Royal College of Physicians and Surgeons of Canada technical reports [17]. These sources often provide timely and contextually rich insights that can inform local program or system improvement but are not easily disseminated to the broader community, and thus, these beneficial resources may be difficult to identify and utilize. We encourage individual programs, specialty-specific committees, and medical training institutions to move beyond internal or technical reporting to take the additional step of broadly disseminating, which may involve publishing in peer-reviewed literature or ensuring access via grey-literature search strategies.

Another important challenge is the limited examples of comprehensive and/or systematic evaluations of CBHPE implementations within and across programs in a given training system. While program and site-specific publications are important, these act as single and limited biopsies of a larger picture; thus, it is not clear how representative these experiences are of a whole implementation, or how generalizable these experiences are to the broader community. Further, there is the potential for gatekeeper effects, whereby the medical education community may be more interested in struggle and in learning how to avoid potential pitfalls, rather than seemingly mundane evaluations describing implementation with fidelity. As such, we encourage implementers and evaluators to become familiar with the CORE-HPE Reporting Guidelines to ensure standardized reporting in CBHPE [91]. We recognize that comprehensive program evaluations require resources, including time, money, and access to expert evaluators for consultation and guidance in study design and analysis, and thus comprehensive extensive program evaluations may not be feasible. We encourage all implementers and evaluators to carefully plan feasible studies that will provide essential insights within the context of available resources.

Although CBHPE has expanded across many countries in the Global South [92], there is still limited published work that evaluates how these implementations are unfolding. Much of this reform has taken place in settings with resource constraints, variable regulatory and accreditation structures, and uneven access to faculty development, yet these contextual realities are rarely captured in current evaluation studies, especially those that are ultimately disseminated via traditional structures. As a result, global narratives about CBHPE are still shaped mostly by experiences from high-income countries, which are often not generalizable to programs in low-resource settings. Strengthening program evaluation efforts in the Global South is therefore essential not only to understand effectiveness and challenges locally but to ensure that global discussions about CBHPE evolution incorporate a broader and more representative range of experiences.

It is important to state that this manuscript was developed by a group of medical education researchers and clinicians representing multiple professional roles, educational systems, and institutional contexts across several countries. Throughout the conception and writing of this manuscript, the authors sought input from all members of the ICBHPE Collaborators Consortium whose members span 6 continents and 14 countries. All members were invited to collaborate, contribute ideas, and ICBHPE Collaborator Consortium members who met the International Committee of Medical Journal Editors (ICMJE) criteria were included as co-authors. Our training, professional interests, and resources incline us toward certain frameworks for implementation, measurement, and interpretation, and we recognize that these orientations may not fully capture the perspectives of educators and learners from other cultural or institutional contexts.

Future efforts should continue to balance methodological rigor with the practicality and “messiness” of implementation, embracing evaluation as an iterative and continuous process rather than achieved in a single evaluation or iteration of implementation. As implementation efforts advance globally, coordinated evaluation strategies, shared frameworks, and broader dissemination will enhance collective learning and strengthen evidence-based CBHPE. Beyond traditional evaluation approaches, frameworks such as *Eco-Normalization* [93] offer a valuable lens for considering the sustainability and contextual integration of innovations across CBHPE. This model builds on the context-mechanism-outcome framework of Realist Evaluation [16], emphasizing the importance of evaluating how an innovation becomes embedded and normalized within its ecosystem, acknowledging that longevity depends not only on effectiveness but also on alignment with institutional culture, resources, and evolving practice contexts. Integrating such perspectives may help programs better assess the sustainability and impact of CBHPE over time, particularly across global settings.

## Conclusion

CBHPE is at various stages of implementation around the globe, with a growing body of program evaluation efforts focused on readiness to implement, implementation fidelity, program assessment validity, and educational and clinical outcomes. The breadth of approaches to program evaluation mirrors the variability in CBHPE structures and processes we see around the globe. By engaging in program evaluation and sharing our findings, be they small-scale or system-wide, we are contributing to the overall understanding of what works and what needs revision, with an aim to provide continued adaptation and evolution of CBHPE programs and frameworks over time.

## AI statement

AI was not utilized in a significant way in the writing or revision of this manuscript.

## Disclaimer

The views expressed herein are those of the authors and not necessarily those of the American Medical Association or any member of the Accelerating Change in Medical Education consortium, the Association of American Medical Colleges, the American Board of Pediatrics, American Board of Pediatrics Foundation, The Royal College of Physicians and Surgeons of Canada, or other Federal or Governmental Agencies.
